# Risk assessment industry driven approach in occupational health and safety

**DOI:** 10.3389/fpubh.2024.1381879

**Published:** 2024-06-04

**Authors:** Katarína Hollá, Alena Kuricová, Samuel Kočkár, Pavol Prievozník, Filip Dostál

**Affiliations:** ^1^Department of Crisis Management, University of Žilina, Žilina, Slovakia; ^2^Faculty of Biomedical Engineering, CTU in Prague, Prague, Czechia

**Keywords:** occupational safety and health, risk assessment, methods, risk matrix, risks, hazards, checklist

## Abstract

**Introduction:**

Risk assessment and management in companies plays a significant role in the prevention section of any field. In the field of Occupational Health and Safety (OHS), its inconsistent or incorrect application has a direct impact on the life and health of employees. In some companies, even today, it is not properly implemented and adequate procedures and methods are not used. The article discusses the development of a step-by-step procedure for risk assessment in industrial environments in the area of OHS.

**Methods:**

Main parts of the model and its steps present the partial results of a survey conducted on a sample of 500 small and micro enterprises in the field of risk assessment and the systematic procedure developed following the main survey results. The survey covered only enterprises located in the construction, manufacturing, transport and storage and agriculture, forestry and fishing sectors, which is also a significant statistical feature. Within the structure of respondents, statistical features such as: size of enterprise, sector, region by work are identified. Only enterprises with size by number of employees - micro enterprises from 1 to 9 employees and small enterprises from 10 to 49 employees - were included for the survey.

**Results:**

New elements of the methods were integrated into the developed systematic procedure, which was subsequently validated in 7 plants of the one company on the same position. The application of the developed model was verified by an expert group consisting of 7 members, an odd number, and the developed checklists and risk register were applied. On the basis of the verification, the model, checklist and risk register were corrected. In addition, the scoring method and the risk matrix were also used, but they did not contain new elements.

**Discusion:**

The procedure is still in use today and employees have been trained to use it. On the basis of the developed methodology and the Checklist, the procedure has been transposed into the European OiRA tool and can be used by companies throughout the European Union.

## 1 Introduction

Risk assessment and management have an indispensable place in OHS for the prevention of occupational accidents. That is why in this article we have decided to devote ourselves to this topic and provide our procedure, selected parts of which were determined by the outputs of a survey whose results are presented in the next section of the article. This risk assessment driven approach has been validated on seven job positions in 7 companies.

The fundamental starting points for risk assessment and management in OHS are as follows:

- There are several **definitions** of risk assessment, but they agree on the following: “Risk assessment and its main objective is to ensure the protection, safety and health of employees. The implementation of a risk assessment minimizes potential harm to employees, the basis of which is prevention” (1).
- All **safety consultants** know that in OHS is one of the most critical step the occupational health and safety risk assessment, which aims to identify, assess, and control high-risk occupational hazards in the workplace for improving the health and safety of workers (2–4).
- Occupational health and safety risk assessment studies have gained importance recently as a result of **increasing occupational accidents and occupational diseases** (5–8). OHS risk assessment studies have recently gained importance as a result of the increase in occupational accidents and occupational disease due to technological developments and the industrialization process. In 2017, there were over 3.3 million non-fatal accidents and 3,552 fatal accidents in the EU-28 Eurostat (8, 9).

Many models and approaches for assessing the risk of occupational hazards have been proposed in previous studies (10). Nevertheless, few contributions are devoted to perform a comprehensive literature review of the researches on occupational health and safety risk assessment (OHSRA). Generally, four stages are included in an OHSRA process: identifying known or potential occupational hazards; determining the causes and consequence of each occupational hazard; evaluating the risk of occupational hazard and providing protection measures; recording important outcomes and reviewing the assessment information. This article should focus on a successful risk assessment procedure in small and medium sized enterprises (SMEs) where occupational health and safety resources may be less accessible than in larger companies with more labor force, time, knowledge and technology (11). In recent years, the OHSRA has attracted extensive attentions from scholars and practitioners, and a growing number of methods have been proposed for improving the health and safety workers in various fields (5).

In the following, we will outline some selected risk assessment approaches and the models developed. One of A new risk-scoring model that combines SWOT analysis with the Hesitant Fuzzy Linguistic Term Set was proposed in article form Adem et al. In this model, SWOT analysis was used, which can be a very good tool (12).

In another article, they recommend a bow-tie diagram for use in determining causes and impacts, which is mainly used for scenario building (12). The use of the Fuzzy approach is very often repeated. This model is adopted to capture the uncertainty and fuzziness of risk evaluations provided by experts (13, 14).

In article (15) other approaches were also presented, but these mainly addressed the challenges associated with MSDs and other risk factors related to occupational diseases. Even more interesting appears to be (16).

The proposed approach is divided into three phases and each phase is divided into steps. This approach outlines all phases of risk management including: (1) risk identification; (2) risk assessment and (3) actions. To address occupational health hazards, the traditional approach is implementing add-on measures to protect workers based on observed hazards in the workplace (17) approaches that are applied in a specific setting and provide valuable information can also be found in articles published by these authors’ collectives (18, 19).

In the Slovak Republic, there are no well-defined rules for risk assessment and subsequent risk management. Due to this fact, some companies do risk assessment inconsistently or external companies that process the documents from the table. That is why by applying our approach we are trying to implement the cooperation of both external expert team and internal team in the application so that employees in the company can learn how to assess risks and then they can implement it themselves in other jobs. We have found that the job roles are suited to the most user friendly and understandable approach so that the people who will need to understand it understand it.

At present, the universality of the approach across multiple job positions and in different environments remains to be validated. Following this, it will be possible to draw further conclusions about its comprehensive use. The basis of the paper is to show the applicability of the developed risk assessment within one job position which was assessed in 7 plants with different production methods. Precisely because there is no access to new methods and models in the Slovak Republic so we decided to create an alternative understandable and verified and especially based on extensive research.

## 2 Methods

The following approaches, methods and techniques were used in the paper, which will be discussed later on:

- Research survey – qualitative method of data collection – Computer Assisted Telephone Interviewing in the Slovak Republic (identification of methods and essential elements that will be part of the model).
- Analysis of existing risk assessment approaches and methods and synthesis of essential features and steps into a developed model (KatAlSA),
- Survey EU-OSHA ESENER 1,2,3.
- Personal consultations with experts in the field of occupational health and safety both in the Czech Republic and the Slovak Republic in the modification of the model and its verification.
- Expert evaluation in risk register method.

In 2023, we conducted a research survey using a qualitative method of data collection – Computer Assisted Telephone Interviewing in the Slovak Republic. The sample size was 500 small and micro enterprises in 4 segments separated on the basis of injury rates namely: construction, industry, transport and agriculture and fishing. The survey was developed according to the international quality standards of WAPOR, ESOMAR and the standards of the Slovak Association of Research Agencies (SAVA), of which AKO is a member.

Due to the fact that the most problematic enterprises in terms of proper implementation of OHS risk assessment are small and micro enterprises, the survey was conducted there. However, what we found was that the below procedure, if applied to a specific job, also works in medium and large enterprises (verification). We conducted the survey using 10 merit, 5 statistical and screening questions. In the following, we report the results of 3 questions that had a direct impact on the development of the KatAlSA risk assessment model.

Using the selected questions, we identified which methods to select for each step of the KatAlSa model.

Questions we were interested in:

- Methods and tools for identifying hazards and threats

“What method or tool do you use to identify hazards and threats in your enterprise?”

**Figure 1:**
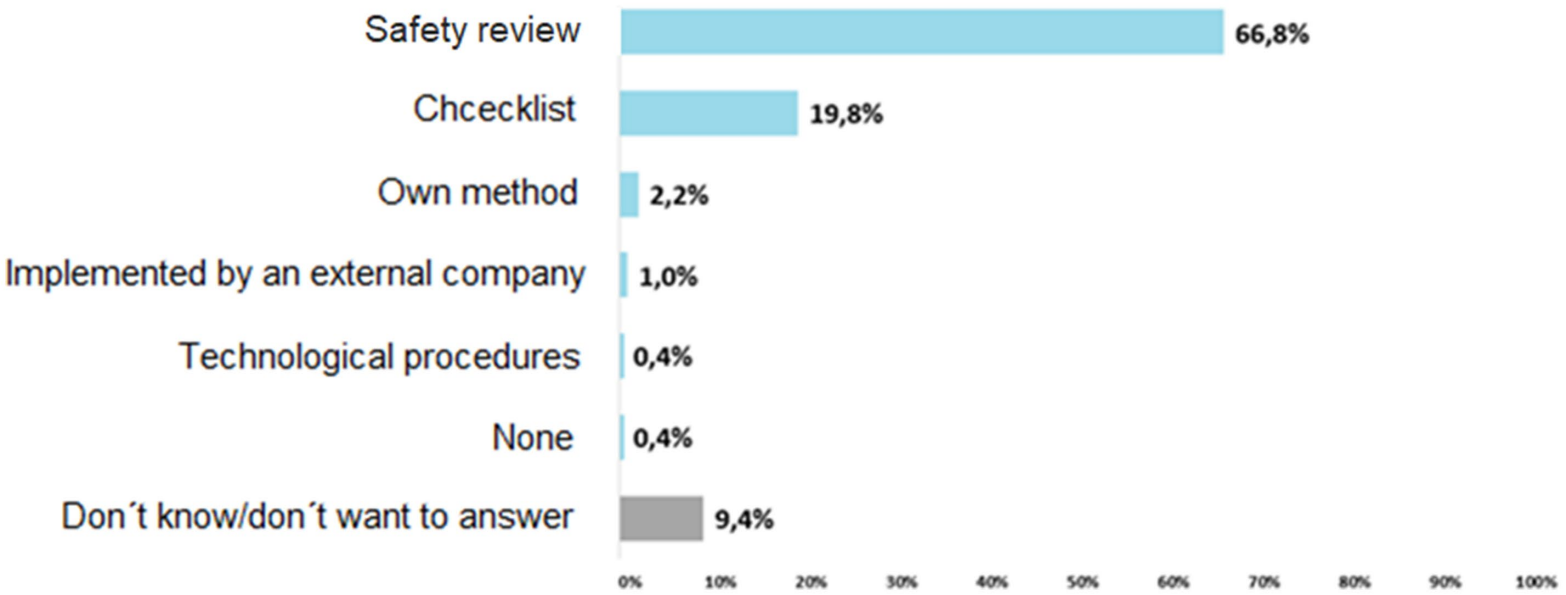
Implemented methods for risk assessment.

The vast majority, i.e., two thirds of the respondents (66.8%) state that they use the **Safety review** method to identify hazards. Compared to the average result of the whole survey sample, the safety inspection is slightly more frequently used (72%) to identify risks in small enterprises (from 11–50 employees) than in micro enterprises (65%). **The Checklist** is used by one-fifth of respondents (19.8%). The checklist is used slightly above average by micro enterprises (22%); from the transport and storage sector (24%) (Figure 1).

In view of this fact, we have decided that Safety review and Checklist will be part of the KatAlSa model.

- The most common safety risk

“Which of the following hazards and risks are most common in your business?”

We used the results of the next question to develop the hazard areas that were included in the checklist.

## 3 Methods and tools for risk assessment of hazards

**Figure 2:**
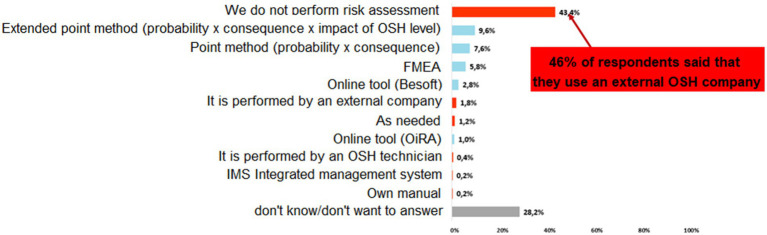
Methods used for risk assessment in enterprises.

“Which method and tool do you use to assess risks from hazards?” (Figure 2).

With this question we needed to find out what method we will use in the risk register (stating probability and consequences). Of the other methods listed, the Extended Points Method (probability x consequence x OHS level impact) and the Points Method (probability x consequence) were in the top two places at 7.6%. It was confirmed that the classical extended scoring method (PxCxI) and the simple scoring method (PxCxI) are the most commonly used and will be implemented into KatAlSa.

**Figure 3:**
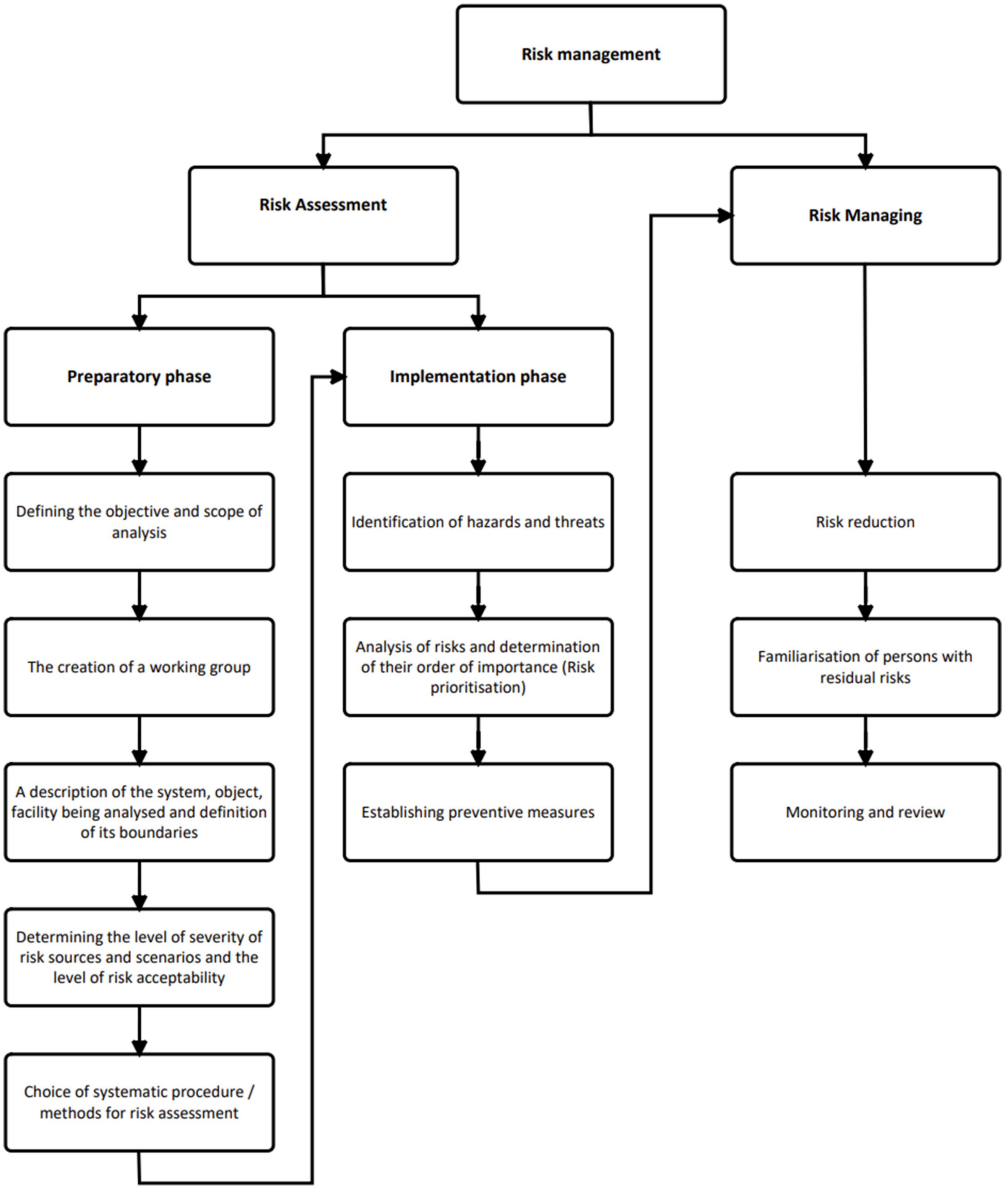
Systematic model of OHS risk assessment KatAlSa.

**Figure 4:**
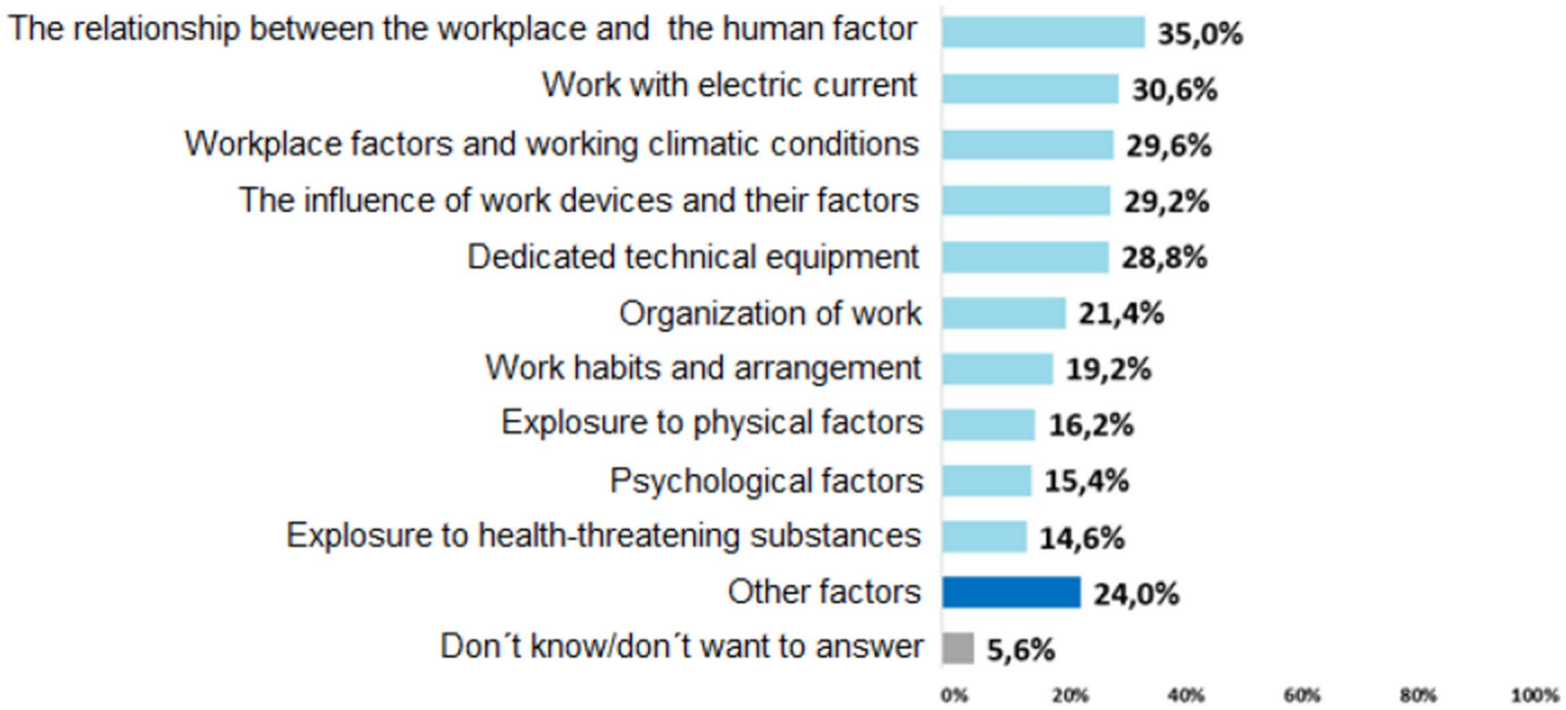
The most common hazards and risks.

The model KatAlSa developed for risk assessment and management in OHS presented in Figure 3 combines two approaches for determining the steps, one developed by Katarína Hollá (Industrial accidents prevention book) and relating to the preparatory phase and the EU-OSHA procedure, which is used by the European Union countries. In the Identification of Hazards and Treats step, a Checklist was implemented based on the areas and issues that were identified in the survey (Figure 4). In the second step of the implementation phase, a Risk register will be used, which includes a risk assessment in two possible alternatives: the PxC and the PxCxI. We propose to determine the acceptability or unacceptability of the risks in a 5×5 matrix.

In this section, we present the information corresponding to each step of the preparatory phase within the KatAlSa model. The implementation phase is presented in the Results section.

**Figure 5:**
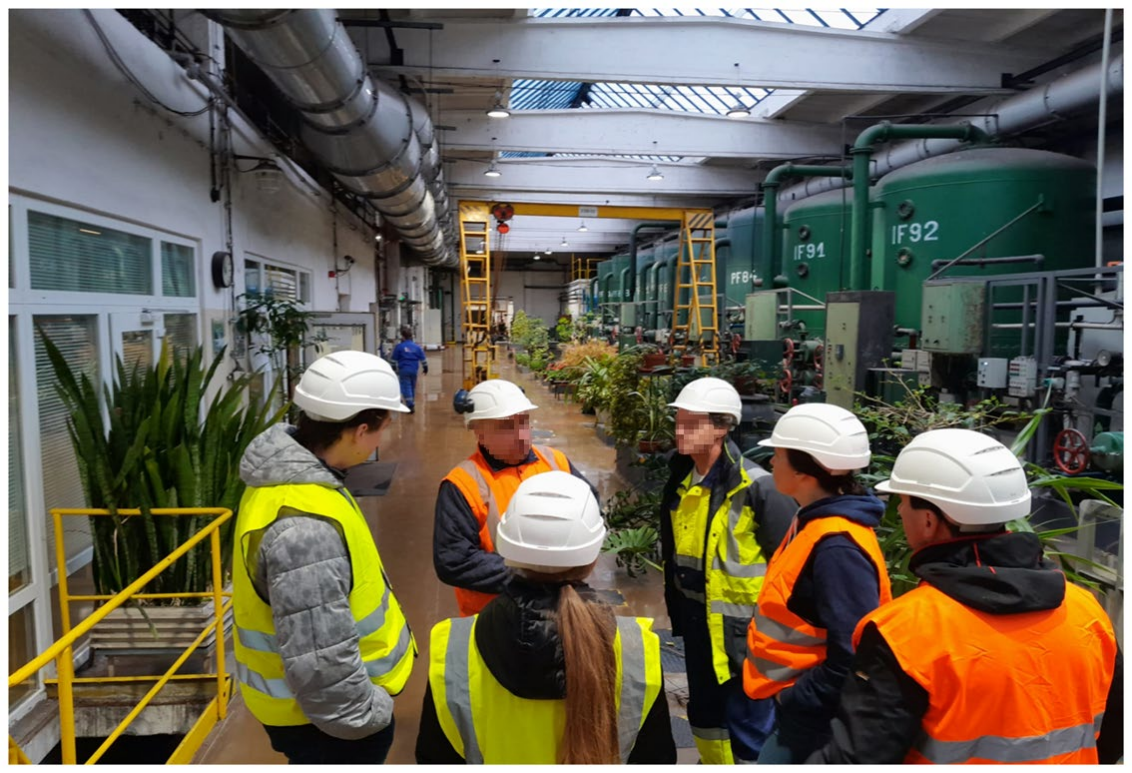
Part of working group on-site in one company.

On Figure 5 shows part of the team directly from the implementation from one plant where the KatAlSa model was applied.

The point of this paper is not to describe in detail the technology and the work position that was analyzed but to point out the results of the implementation of the KatAlSa model method and to verify the developed this systematic procedure. This application was implemented in 1 year horizon.

We will follow all the steps from preparatory phase of the KatAlSa model (Figure 3).

### 3.1 Determination of the objective and scope of the analysis

The intention was to standardize the risk assessment process across the plants and to carry out a pilot application at a selected workstation in all plants. The requirement was to meet the requirements of the Occupational Health and Safety Act in the Slovak Republic as well as a user friendly fist for the employees who will continue to do this in the company.

### 3.2 Creation of a working group

The first part of the working group consisted of a team from the University of Žilina. There were two experts within the UNIZA team who were able to apply created checklist and Risk register in detail, and they led the application of these two methods in a face-to-face meeting. Other persons studied the technology in detail and inquired the necessary information from the employee in case they were need. Plus, in each plant, the safety engineer, foreman, HSE manager for the whole holding and a workplace representative became part of the working group. Each assessment was carried out first by a safety review and documenting everything needed directly for the job location, followed by an expert assessment in a meeting room using all together brainstorming session.

### 3.3 Description of the object to be analyzed and determination of its boundaries

Company XY, interconnects and streamlines the activities of seven heating companies. They provide thermal comfort and services to households and residents. We focused on the job position of Machinist.

The position of Machinist plays a key role in the operation of a heat production plant. This position requires the expertise and skills to manage process equipment in the preparation and implementation of energy production and conversion. The Machinist is responsible for compliance with the Operations and Maintenance Manual, Organizational Safety Rules and Emergency Procedures.

As part of his/her professional activities, the Machinist is involved in the maintenance and operation of equipment at the assigned operating site. His/her task is to carry out inspection and preventive activities in order to maintain the established operating parameters of the technological equipment. This includes the maintenance and inspection of various filters such as catex, anex, and mix ion filters, with an emptying and inspection at least once a year. In addition, the water engineer is involved in the maintenance and inspection of the filtration equipment, with the emptying and inspection of the sand filter of the water after clarification taking place once every three years. He also takes care of the maintenance of the sand filter for the return condensate every two years. In addition to the filters, he also cleans and revises the sludge and emergency pits, performing these tasks as needed, several times a year. The overall maintenance and inspection also includes cleaning and inspection of the technical river water inlet from silt and sediments, cleaning and inspection of clarifiers and storage tanks of operating chemicals. These activities are carried out according to a set schedule and individual shifts. The Machinist also performs cleaning, inspection and maintenance work, working approximately 40% of the annual Working Time Fund. In addition to physical maintenance, he/she is also involved in administrative tasks, including making records and reports in designated operational documentation relating to the operation and maintenance of the facilities.

The Machinist is active in coordination with other trades in the shutdown, start-up, inspection, and testing of equipment within the designated work area. In addition, he/she makes suggestions for carrying out servicing and repair work on the equipment in operation and is also responsible for the operation of pressure vessels. Overall, it follows that the Machinist in a heat production enterprise plays a critical role in ensuring the reliable operation of process equipment and compliance with heat production standards and regulations.

### 3.4 Determination of the level of severity of sources of risk

**Figure 6:**
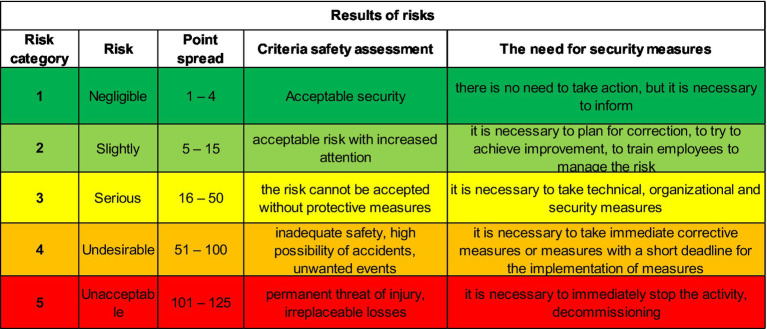
Risk matrix for the extended point method.

**Figure 7:**
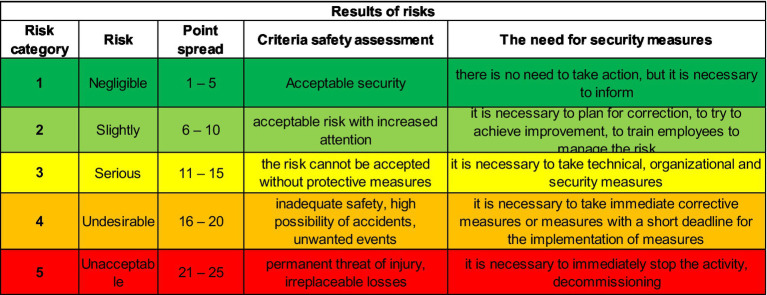
Risk matrix for the simple point method.

In the step of identifying hazards and threats, the selection is just a yes or no answer. If the answer is yes, the hazard associated with the threat is there and is copied into the risk register and subjected to further analysis. In the case of the point method, the criteria are shown in Figures 6, 7.

### 3.5 Selection of systematic risk assessment procedure/methods

We created a new procedure based on existing approaches but with customized elements implemented into certain steps. We call it KatAlSa beginning with the first letters of team who created it.

## 4 Results

The developed model KatAlSa was implemented on one work position in 7 plants within XY Holding focused on heating production. Individual plants have different heat production methods. One of the riskiest positions is the Machinist, which will also be the subject of the following risk assessment. We followed the systematic procedure of KatAlSa and the aim was also to verify the Checklist and Risk Register created. In this section we will show the main results of Implementation phase.

### 4.1 Hazards identified in each plant in XY Holding

**Figure 8:**
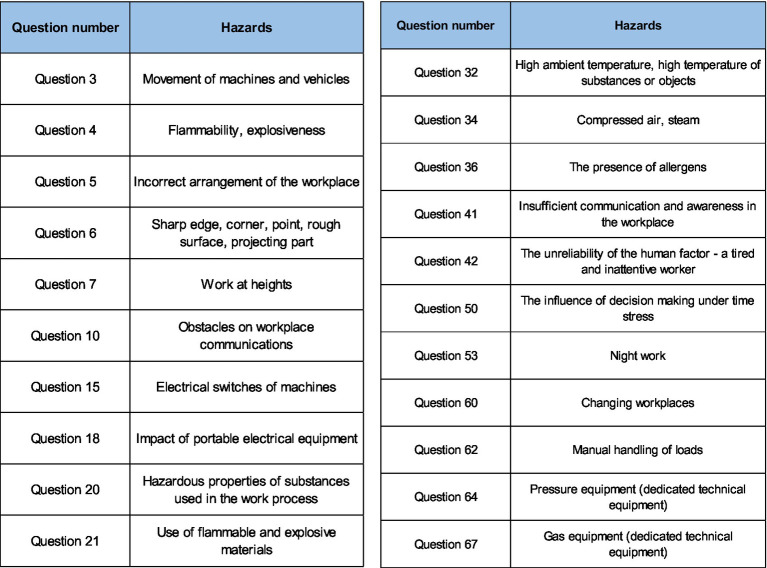
The most common risks in the job position of Machinist.

In the next 2 (Figure 8) tables we list the most frequently occurring hazards that were identified for all 7 positions, based on the hazards listed in the Checklist that was created and implemented into the KatAlSa model.

**Figure 9:**
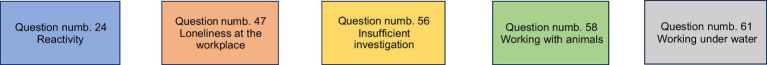
Hazards that do not occur in factories for the position of Machinist.

Figure 9 shows the hazards that were not identified at any of the plants.

The following text shows the results of the expert risk assessment of each plant for the assessed position of Machinist, top 5 risks.

**Figure 10:**
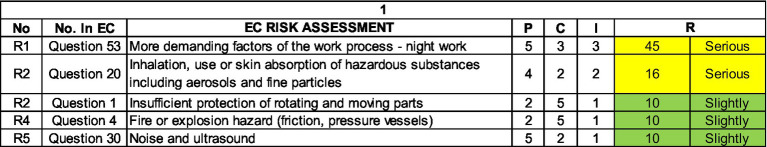
Top 5 risks in the plant 1 within the job position Machinist.

**Figure 11:**
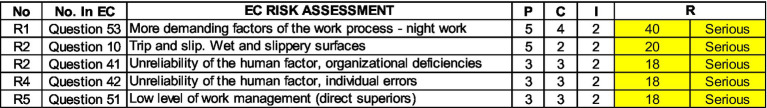
Top 5 risks in the plant 2 within the job position Machinist.

In plant 1 (Figure 10), plant 2 (Figure 11) and plant 3 (Figure 2), the biggest risk is night work, which has been the most significant in terms of downsizing in these plants in particular, and is now a significant issue in the plants and needs to be addressed by management. In plant 1 (Figure 10), plant 2 (Figure 11) and plant 3 (Figure 2), the biggest risk is night work, which has been the most significant in terms of downsizing in these plants in particular, and is now a significant issue in the plants and needs to be addressed by management.

Human factor failure permeated all enterprise applications, and we identified the potential for error in individual and collective mistakes as well as mistakes caused by poor decisions or working under stress.

**Figure 12:**
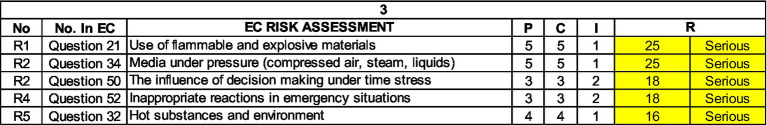
Top 5 risks in the company plant 3 within the job position Machinist.

**Figure 13 fig13:**
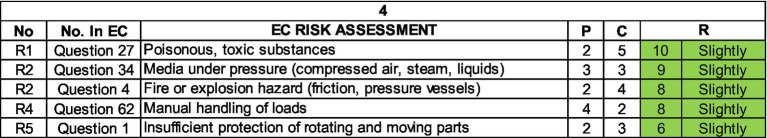
Top 5 risks in the plant 4 within the job position Machinist.

**Figure 14:**
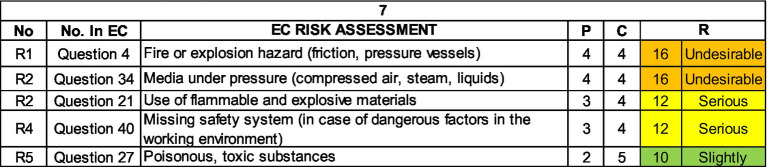
Top 5 risks in the plant 7 within the job position Machinist.

Working with hazardous substances is another of the significant hazards in this undertaking and in this job with the potential for fire, explosion or spillage of a hazardous substance. The identified hazards may also have implications for occupational diseases caused by prolonged exposure to lower levels of hazardous substances (Figures 12 [Fig fig13]–14).

**Figure 15:**
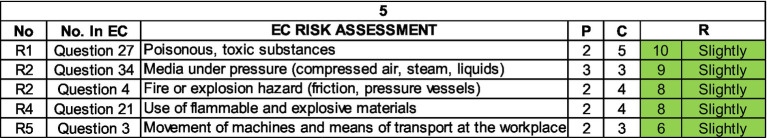
Top 5 risks in the plant 5 within the job position Machinist.

**Figure 16:**
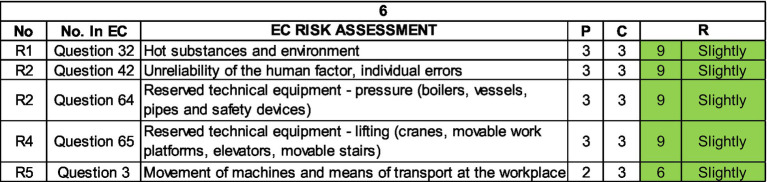
Top 5 risks in the plant 6 within the job position Machinist.

At workplaces, the movement of machinery and equipment was also an identified hazard, which poses a problem in terms of minor injuries or moderate injuries which we identified in the accident book (Figures 15, 16).

## 5 Discussion

The application in all plants for selected positions lasted one year. During the application, the checklist questions were modified so that the plant staff could apply the checklist when it was updated. Other questions were added as needed with the expert team. Another important result was that we were directly with the affected employees for each application and then trained them after the work was handed over.

The methodology we created with a precise procedure and application layout was useful. A risk register checklist was created in an excel file. Furthermore, a complete Risk Assessment report was created for the position of Machinist.

Risk Assessment report was built over all collected data that were structured into a DataCube with core dimensions:

- Job position
- Plant
- Risk level

It is important for users who will use the model in practice to follow certain requirements:

- Compliance with the legal requirements that are imposed on the proposed model.
- Risk assessment must be done with certain members of the company (safety technician, company holder, senior staff member…)
- Carrying out regular site visits.
- Analytical thinking in the process of implementing the model.
- Receiving feedback from employees.

During the verification of the KatAlSa model, a high level of universality was found. The model was initially planned and designed for small and micro enterprises, however, the model can be applied in large companies. No limitation in the proposed model was identified during the verification process that would indicate model errors. We can confirm that the model is applicable and suitable for companies for the further application for jobs.

As an added value we created report system model Based on aggregation of the data through the dimensions. These templates were filled with real data as requested through several iterations from the application of KatAlSa. Finally, an interactive reporting interface was designed and developed in MS Excel office application.

**Figure 17:**
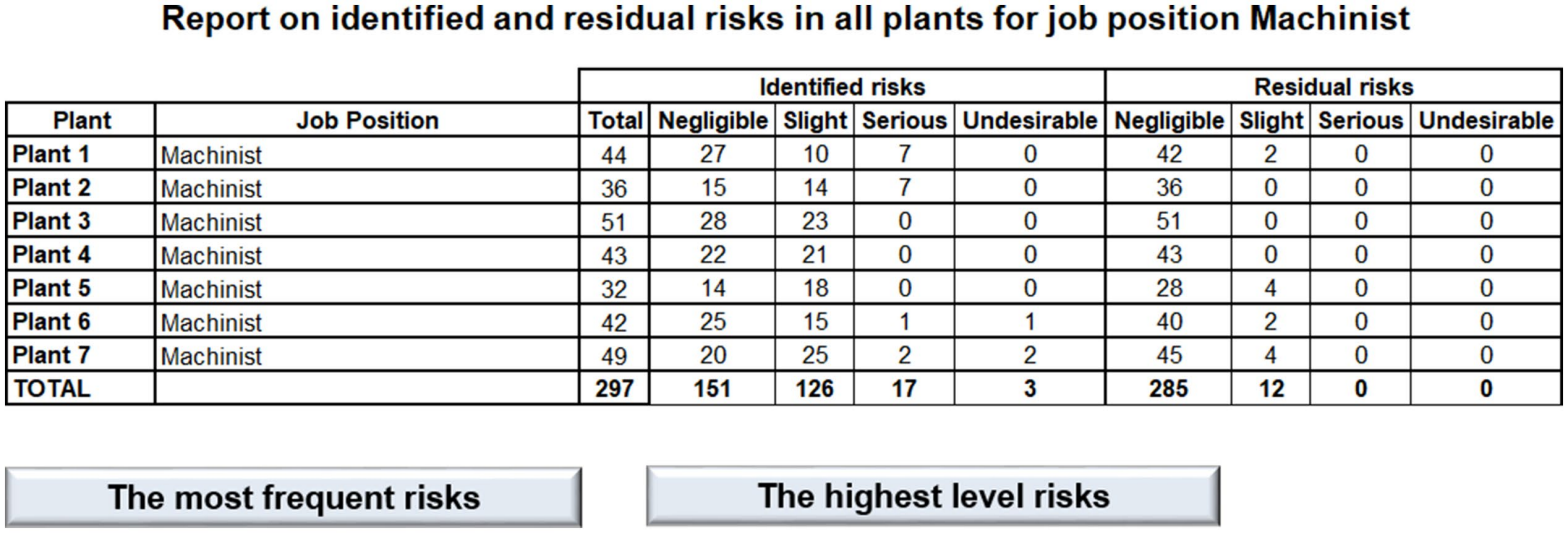
Report on identified and residual risks.

Main Node (Global View) reports data on the Holding as a whole (see the next Figure 17). Structured data are reported as an information table on the number of risks of various levels based on the point method classification for all plants. Specific information on each plant is available through a hyperlink to the 1st level nodes (visualized as buttons). Further, detailed information on the most frequent risks, and the highest level risks throughout all the 7 plants are available through a hyperlink to the 2nd level node in which a table summarizes related information.

**Figure 18:**
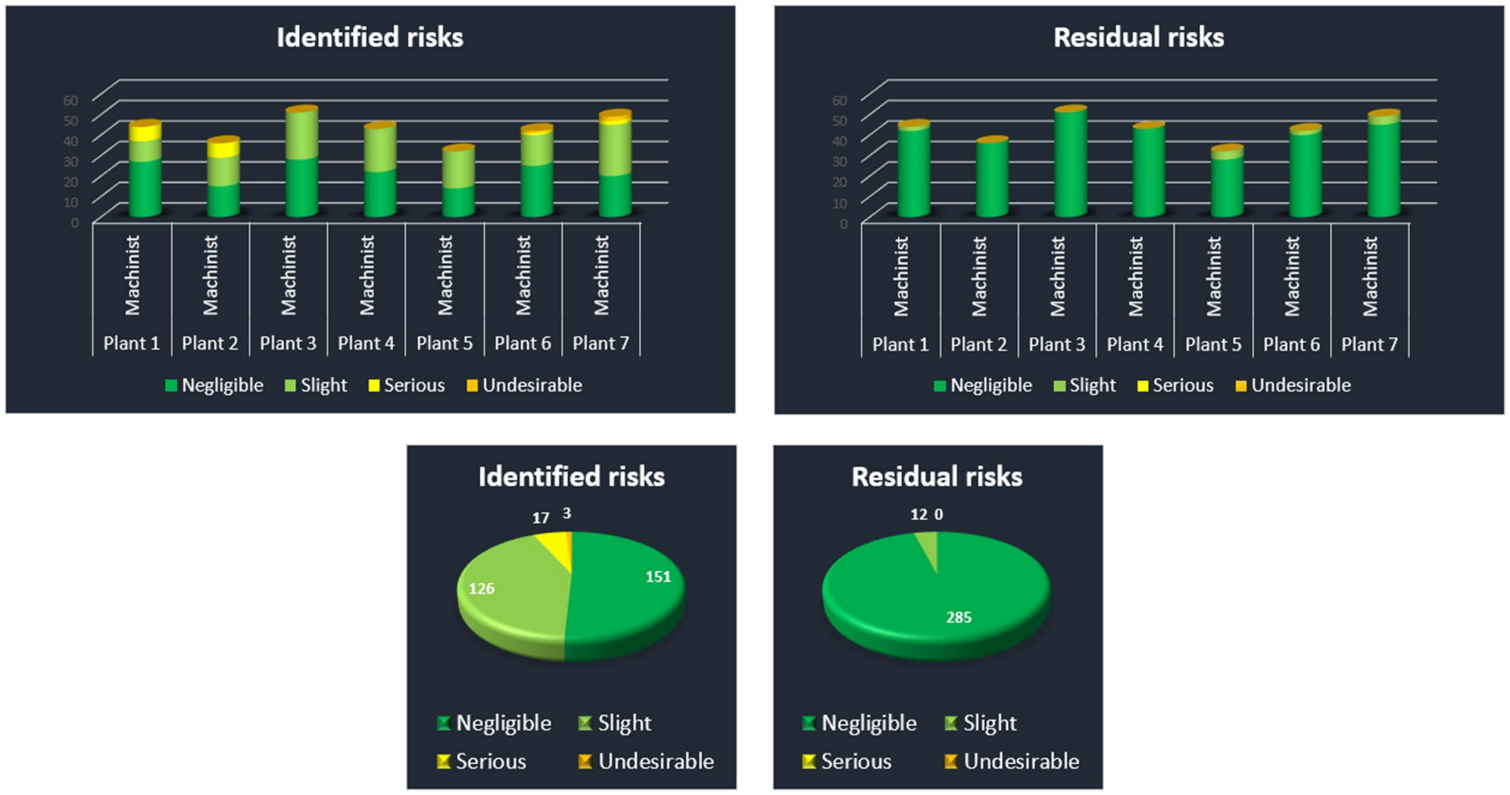
Identified and residual risks.

In addition, the table view on the risk reduction effect is supplemented by graphical representation of identified risks vs. residual risks in column graphs as well as in pie graphs (see the next Figure 18).

**Figure 19:**
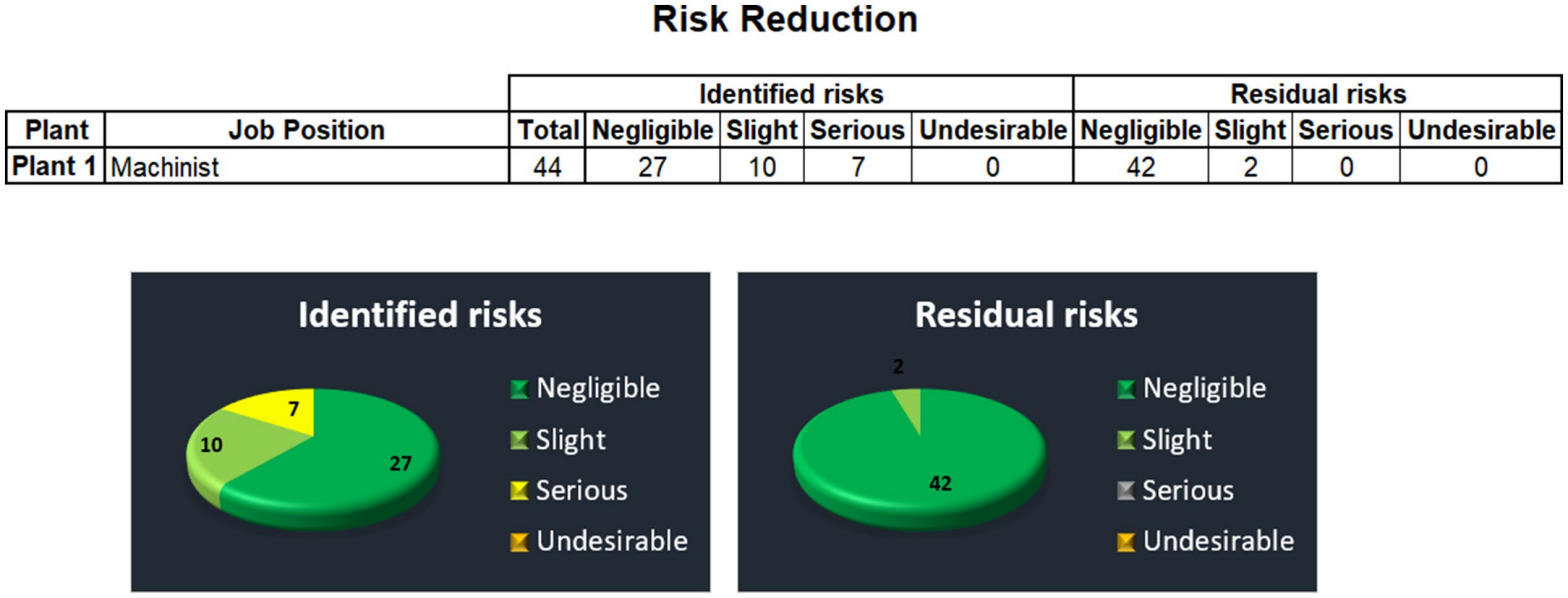
Risk reduction.

1st Level Node (Plant View) reports on data on particular plant within the Holding. These reports are similarly structured as the Main Node report (see the next Figure 19). It has the same navigation panel on the top which is followed by very brief table of the numbers of identified and residual risks to be compared to illustrate the effect of risk reduction in the particular plant. Also graphical representation of the table is offered bellow the table.

2nd Level Node (Detail): As we have moved to the 1st level of the reporting structure, more specific details are provided to the user. Just by clicking on the pie graphs, the user is moved through a built in hyperlink to the complex table of all identified risks, measures to be taken, and residual risks for this particular plant.

This interactive reporting user interface provides OHS managers with a tool to share information on risks, including risk assessment of the current state, measures that need to be taken, as well as the risk assessment of the desired state after the measures will be taken. It is possible to share this information through company’s intranet, and make the information on risk assessment available to any person that is involved in OHS management. Hence, utilizing this management tool increases the OHS level in the organization. The results show that utilizing application of risk assessment on the Holding can bring about essential risk reduction which in fact reduces the expected extent of damage, and hence the expected cost of recovery after any emergency event that would arise from identified hazards.

Specifically, the report says that the number of undesirable risks would be reduced from 3 to 0, the number of serious risks decreases from 1 to 0, and the number of slight risks is reduced from 126 to only 12. At the end, the number of negligible risks increases to 285 leaving only 12 slight risks to be dealt with by the management.

Repeated visualization of repeated risk assessment has the potential to provide management with current data as a supporting tool to reduce expected costs on OHS related accidents.

**Figure 20:**
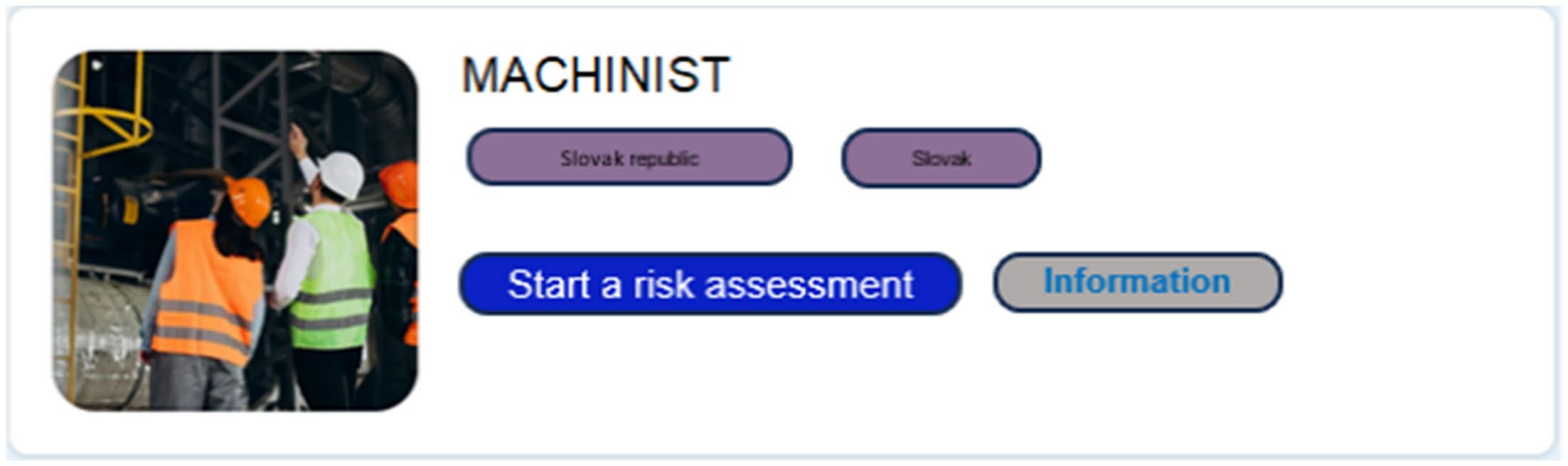
The tool Machinist in the OiRA webplatform (20).

In the last step, we decided to implement the procedure in the OiRA tool, which is intended for micro-enterprises and small enterprises, but the job position of Machinist is also in those enterprises (20) (Figure 20).

In further research, we want to look at the application of the model in other companies and its customization for the selected job position. We are already applying it in another enterprise and the individual questions are adapted to the job position. Also, the reporting system still needs to be improved in terms of visualization and user friendly environment. After applying it in the cement plant to a completely different work position, we will be able to customize the model even further. Already with the current application, I can say that the current clarity of the hazard checklist is sufficient and we can consider it validated and usable for the next field. We have added 4 more questions/hazards into the checklist in the expert evaluation, and the number of questions has increased to 85.

## 6 Conclusion

During the verification of the model, a high degree of universality was found. The model was initially planned and designed for small and micro enterprises, however, the model was verified and implemented in a large enterprise in 6 plants and 7 plants. We can confirm that the model is applicable and suitable for enterprises for further application for jobs. One of the limitations of the model is that it may seem complex for micro-enterprises. In view of this fact, we have also created a simplified version that should be applicable step-by-step also in these enterprises. It is also possible to see that some companies are comfortable with risk evaluation, which consists of probability and consequences, and some prefer to take into account the impact of the level of safety (3 factors).

The KatAlSa model represents a significant step forward in the field of OSH, risk assessment and OSH culture. The model is innovative as it is designed on the basis of the latest available information on risk assessment, either in Slovakia or in the European Union, statistical surveys from the Slovak Republic and major European ESENER surveys 1,2,3, on the basis of which countless manufacturing companies have been surveyed. The model is also significant on the basis of knowledge and experience from practice, experience from experts in the field of OSH in the Slovak Republic and the European Union, information obtained through a questionnaire survey from respondents in the field of OSH of specific sections according to SK NACE and which was also carried out to confirm or refute the proposed hypotheses of the dissertation. The benefits of the model on its applicability in practice for any job position were also confirmed by OSH managers, OSH specialists who have been working in OSH fields for many years and have extensive experience in assessing and managing risks in the areas of construction and industrial production in unnamed construction plant.

## Data availability statement

The raw data supporting the conclusions of this article will be made available by the authors, without undue reservation.

## Author contributions

KH: Conceptualization, Formal analysis, Methodology, Supervision, Writing – original draft, Writing – review & editing. AK: Data curation, Investigation, Validation, Visualization, Writing – original draft. SK: Data curation, Software, Validation, Visualization, Writing – original draft. PP: Data curation, Investigation, Project administration, Writing – review & editing. FD: Formal analysis, Investigation, Writing – review & editing.
